# Growth Rate of *Escherichia coli* During Human Urinary Tract Infection: Implications for Antibiotic Effect

**DOI:** 10.3390/antibiotics8030092

**Published:** 2019-07-12

**Authors:** Maria Schei Haugan, Frederik Boëtius Hertz, Godefroid Charbon, Berivan Sahin, Anders Løbner-Olesen, Niels Frimodt-Møller

**Affiliations:** 1 Department of Clinical Microbiology, Rigshospitalet, 2100 Copenhagen, Denmark; 2 Department of Biology, University of Copenhagen, 2100 Copenhagen, Denmark; 3 Department of Clinical Microbiology, Herlev Hospital, 2730 Herlev, Denmark

**Keywords:** chromosome replication, bacterial growth rate, antibiotic effect, urinary tract infection

## Abstract

*Escherichia coli* is the primary cause of urinary tract infection (UTI), which is one of the most frequent human infections. While much is understood about the virulence factors utilized by uropathogenic *E. coli* (UPEC), less is known about the bacterial growth dynamics taking place during infection. Bacterial growth is considered essential for successful host colonization and infection, and most antibiotics in clinical use depend on active bacterial growth to exert their effect. However, a means to measure the *in situ* bacterial growth rate during infection has been lacking. Due to faithful coordination between chromosome replication and cell growth and division in *E. coli*, chromosome replication provides a quantitative measure of the bacterial growth rate. In this study, we explored the potential for inferring *in situ* bacterial growth rate from a single urine sample in patients with *E. coli* bacteriuria by differential genome quantification (*ori:ter*) performed by quantitative PCR. We found active bacterial growth in almost all samples. However, this occurs with day-to-day and inter-patient variability. Our observations indicate that chromosome replication provides not only a robust measure of bacterial growth rate, but it can also be used as a means to evaluate antibiotic effect.

## 1. Introduction

The urinary tract constitutes the most common site of human bacterial infection, and *Escherichia coli* is, by far, the most prevalent causative organism at this site [1,2]. Most urinary tract infections (UTI) result from ascension of bacteria from the urethra to the bladder, and possibly kidneys [3]. Bacterial growth is considered essential for evasion of the host immune response and successful establishment of infection [4]. Furthermore, bacterial growth is critical for most antibiotics in clinical use to exert their effect [5,6,7,8,9,10,11,12]. While much is understood about the virulence factors utilized by uropathogenic *E. coli* (UPEC), less is known about the bacterial growth dynamics taking place during human infection [13,14]. To date, there exists no gold standard method for probing the bacterial growth rate during a host infection. Extracting bacterial growth rates from bacterial count kinetics is convenient *in vitro*, but not during human infection where frequently repeated sample measurements are not feasible, and the contribution of the host immune system to bacterial elimination is not considered. However, in recent years, as complete bacterial genome sequences have become broadly available, it has been possible to probe in-host bacterial growth rates by differential genome coverage analyses from either whole-genome sequencing [15,16,17,18] or quantitative PCR (qPCR) data [19]. These methods are based on the principle that growth of *E. coli*, like many other bacteria, is precisely coordinated with the replication of its single circular chromosome [20]. In *E. coli,* growth-dependent chromosome replication is initiated from a single defined origin of replication (*oriC*), from where replication is carried out bidirectionally by two replication forks moving toward the opposite located terminus of replication (*terC*) once per cell cycle [20,21]. Given beneficial growth conditions, the bacteria grow with overlapping replication cycles, where chromosome replication is initiated synchronously from 2*^n^* (*n* = 1, 2, 3) origins, which is a phenomenon termed multifork replication [22]. This allows for doubling times shorter than the replication time. Hence, the copy number of chromosome *oriC* relative to *terC* (*ori:ter* ratio) is positively correlated with bacterial growth rate, which we have recently demonstrated in the murine peritonitis/septicemia model [19]. As a reference, when measured in *E. coli* ATCC 25922 under controlled growth *in vitro*, an *ori:ter* of ~3 represents maximal growth rate (i.e., a doubling time of approximately 20 min) and an *ori:ter* of ~1 represents minimal growth rate (i.e., no growth) [19].

In this case, we extended the approach of inferring *in situ* bacterial growth rate from a single biological sample into exploring its potential for use in human urinary tract infection (UTI). We also aimed at testing *in vivo* antibiotic effect by differential genome quantification measurement. Patients both with and without (i.e., asymptomatic bacteriuria) symptoms of UTI were included to test for possible differences in growth dynamics. Urine samples were collected daily for up to four days to evaluate the temporal development in the bacterial growth rate.

## 2. Results

### 2.1. Study Population 

**Figure 1:**
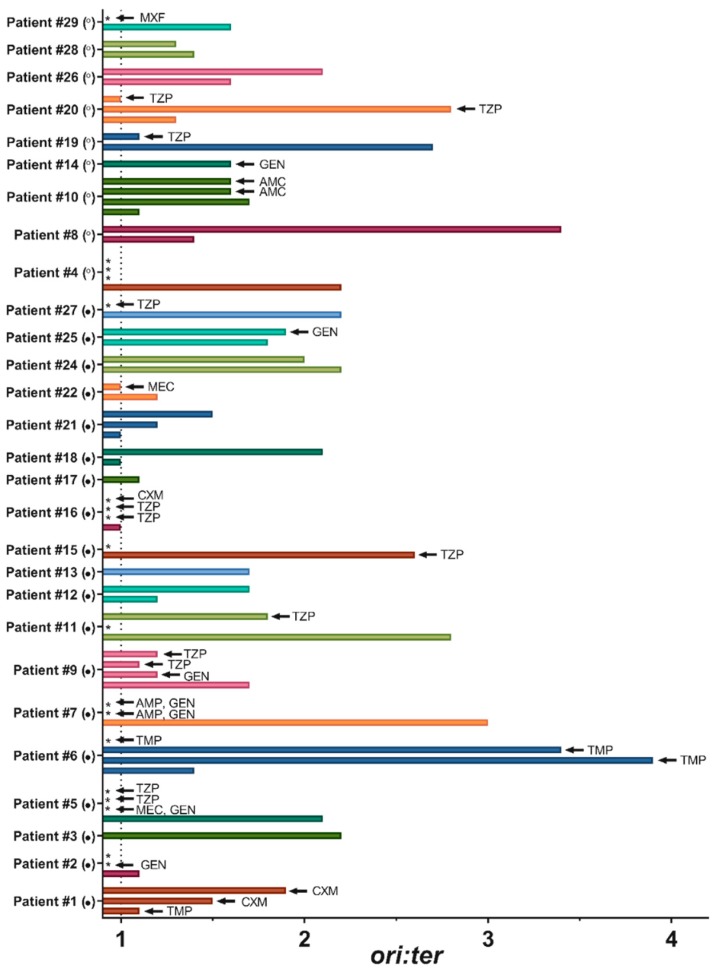
Distributions of *ori:ter* in the study population. Each bar represents the mean *ori* copy number of technical triplicates relative to the mean *ter* copy number of technical triplicates analyzed from one urine sample. For every patient, the lower bar represents the day 0 urine sample, and the follow-up urine samples (day 1 up to day 3) are presented in upwardly directed chronological order. Urine samples not yielding adequate copy numbers for quantification are represented by an asterisk. The arrow indicates that antibiotic treatment (any relevant) had been given at the time of urine sampling. No relevant antibiotic treatment had been given at the time of urine sampling. The closed circle indicates that the patient was classified as having UTI. The open circle indicates that the patient was classified as not having UTI. The dotted line represents the minimum possible *ori:ter* level (i.e., no growth). Patient no. 23 is not included, as no *ori:ter* was available. AMC: amoxicillin-clavulanic acid. AMP: ampicillin. CXM: cefuroxime. GEN: gentamicin. MXF: moxifloxacin. TZP: piperacillin-tazobactam. PMC: pivmecillinam. TMP: trimethoprim.

In this study, a total of 31 hospitalized adult patients with significant quantities of *E. coli* bacteriuria were included. Two patients were later excluded with one due to retraction of patient consent and one due to revision of the preliminary microbiological identification result. From the remaining 29 patients, the initial urine sample originally sent to the Department of Clinical Microbiology for culture (day 0) was collected (Figure 1, Table S1). Subsequent follow-up urine samples on day 1, day 2, and day 3 were provided from 25, 12, and 6 patients, respectively (Figure 1, Table S1). 

In total, 72 urine samples were, therefore, available for analysis. Patient characteristics are outlined in Table 1. In brief, the majority were geriatric (69% were >70 years old), female, suffering from competing illnesses, and had a short duration of symptoms (if any) of urinary tract infection. Five patients had disseminated infection (*E. coli* bacteremia). Only three patients received relevant antibiotics (prophylactic) before collection of the day 0 urine sample, whereas the majority received antibiotics after collection of the day 0 urine sample, which is either for suspected UTI or for other infection (Table S1, Figure 1). Almost all patients receiving antibiotic treatment received combination therapy and/or changing regimens.

### 2.2. Bacterial Growth Rates in Human Urine

Bacterial growth rates, expressed as copy number quantification of *oriC* relative to *terC* (*ori:ter*) by qPCR, were successfully inferred from the day 0 urine sample from 28 out of the total 29 patients (Figure 1, Table S1). From the 23rd patient, the volume of urine provided was too low (< 1 mL) for adequate DNA purification (Table S1). We were able to detect *ori:ter* ratios in most, but not all, follow-up urine samples on day 1–3, due to successful elimination of bacteria in the urine after relevant antibiotic treatment, as confirmed by the absence of regrowth on selective agar plates (patients no. 2, 5, 6, 7, 15, 16, 27, and 29, Figure 1, Table S1). In patient no. 4, who was classified as not having UTI, bacteria were eliminated after day 0 (confirmed both by the lack of qPCR amplification and the lack of regrowth) without antibiotic treatment, which is suggestive of transient asymptomatic bacteria (Figure 1, Table S1). In a few other follow-up urine samples (no. 11-1, 16-1, 16-2, and 23-1, Figure 1, Table S1) the volume of urine provided was too low for adequate DNA purification. Additionally, in other follow-up urine samples (no. 1-1, 1-2, 10-2, 10-3, 20-1, 20-2, and 22-1, Figure 1, Table S1) we were able to quantify *ori:*ter, without regrowth on selective agar plates. This phenomenon is likely due to antibiotic carry-over effect, as all samples were collected after one or more days of adequate antibiotic therapy [23]. With only a few exceptions, there was overall active bacterial growth (i.e., *ori:ter* > 1), with a median (range) *ori:ter* ratio of 1.6 (1–3) in the day 0 urine samples, and a median (range) *ori:ter* ratio of 1.65 (1–3.9) in the follow-up urine samples (day 1–3). 

Follow-up urine samples from 18 out of 25 patients were taken after antibiotic exposure. Hence, the *ori:ter* ratios observed in these bacterial populations could be affected by the antibiotic given. This phenomenon is exemplified in patient no. 6, which presents the highest *ori:ter* level observed in all urine samples available (Figure 1, Table S1) and higher than any levels previously reported in human urine [15]. In this case, a substantial increase in *ori:ter* following exposure to therapeutic doses of trimethoprim could be explained by a successful reduction of the nucleotide pool [24], which interferes with ongoing chromosome replication and, thus, prevents replication forks from reaching the terminus [20]. This phenomenon, followed by the eradication of bacteria (as demonstrated both by the lack of regrowth and the lack of qPCR amplification in the last follow-up urine sample from this patient, Figure 1, Table S1), indicates that treatment was efficient. In patient no. 1, however, trimethoprim was given as prophylactic therapy before collection of the day 0 urine sample (Figure 1, Table S1). Despite *in vitro* susceptibility toward the drug, the patient presented with disseminated infection and an *ori:ter* ratio close to 1 are both indicative of treatment failure. To what extent the *ori:ter* levels probed from urine samples following exposure to antibiotics with other cellular targets represent true bacterial growth rates, or whether these *ori:ter* levels were affected by the antibiotic in question, is less certain. We have, however, previously observed a decrease in *ori:ter* following exposure to β-lactam antibiotic (ceftriaxone) or aminoglycoside antibiotic (gentamicin) during experimental murine infection, due to preferential elimination of fractions of rapidly growing bacteria [5]. A similar decrease in *ori:ter* following exposure to various β-lactam or aminoglycoside antibiotics was observed in the majority of patients providing follow-up urine samples with quantifiable *ori:ter* levels (patients number 9, 11, 22, 10, and 19, Figure 1, Table S1). This is suggestive of antibiotic treatment effect. Only in the 25th patient, a marginal increase in *ori:ter* following relevant exposure to gentamicin was observed, and, in the 20th patient, there was an initial increase, prior to a substantial decrease, in *ori:ter* following relevant exposure to piperacillin-tazobactam (Figure 1, Table S1). 

We were able to track natural development in bacterial growth rates (i.e., *ori:ter* in the absence of antibiotics) over time in several patients. Natural growth dynamics in patients classified as having UTI and not having UTI are exemplified in patients number 12, 21, and 24 and in patients number 8 and 26, respectively (Figure 1, Table S1). There was overall active bacterial growth in both groups (i.e., *ori:ter >* 1 in all patients) with day-to-day and patient-to-patient variability. In all but one patient, there was a temporal increase in *ori:ter*. It is not clear whether the observed inter-patient variability in the bacterial growth rate could be attributed to host factors (e.g., age, comorbidity, urine composition, competition from other bacteria, or presence/duration of urethral catheter, Table S1), or whether it could be strain-specific.

### 2.3. Bacterial Growth Rates During Controlled Propagation (in vitro)

To determine whether the inter-patient variability in *ori:ter* ratio observed in the urine samples could be strain-specific, we randomly selected five different patient isolates (from patients number 1, 6, 10, 16, and 29) to undergo growth in a controlled medium with a defined inoculation time *in vitro*. We selected isolates from patients with and without UTI to test whether any difference in growth rate could be due to possible different pathogenic potential of the isolate. *E. coli* ATCC 25922 was grown in parallel as a control. The clinical isolates were subject to whole-genome sequencing. From these data, we were able to distinguish the isolates by serotype and estimated the genome size. All five clinical isolates were confirmed as *E. coli* and all were *fimH* positive, which allows for possible expression of type 1 fimbriae required for successful colonization of the urinary tract [25]. None of the isolates were identical. Isolate number 1 was identified as serotype O2:H1, number 6 as O6:H1, and number 16 as O8:H25. Estimated genome sizes were 5.2, 5.0, and 4.9 Mb, respectively. The O antigens identified in these strains belong to the most frequent O antigens observed in UPEC [26,27], and all three patients were classified as having UTI (Table S1). Isolate number 10 and 29, however, originated from patients classified as not having UTI and were identified as serotypes O166:H15 and O8:H10, respectively (Table S1). The estimated genome size of both was 4.9 Mb. 

**Figure 2:**
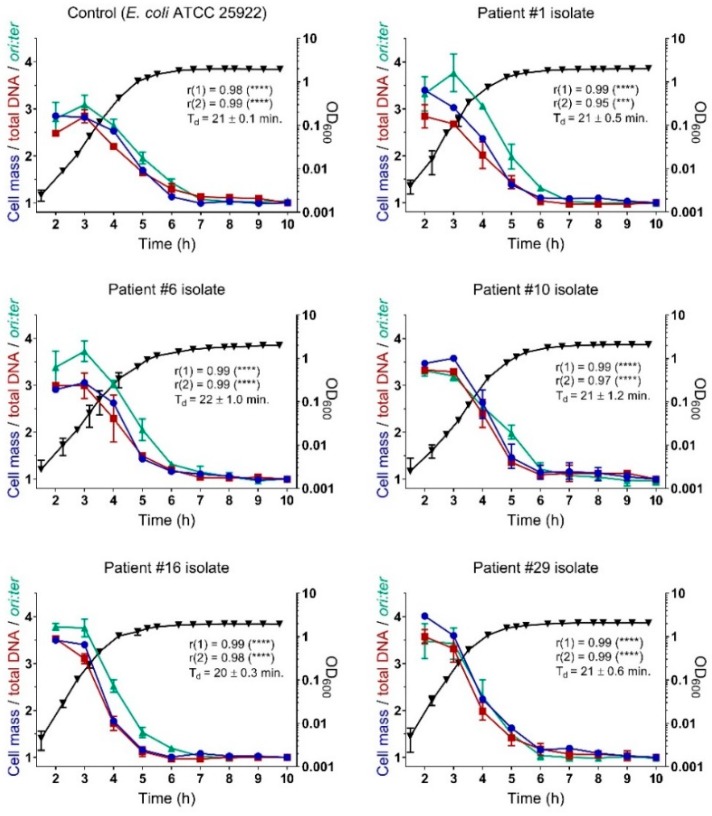
Controlled growth of selected clinical *E. coli* urine isolates in Lysogeny Broth (LB) batch cultures *in vitro. E. coli* ATCC 25922 was used as a control. Bacterial growth measured as optical density (OD_600_) (black triangles), *ori:ter* (green triangles), cell mass (blue circles), and total DNA content per cell (red squares). The latter two measurements were made by flow cytometry and are presented as relative to those of the same isolate during the late stationary phase (i.e., the samples collected at 10 h of incubation). An average of 30,000 bacterial cells were analyzed by flow cytometry per sample. Data are presented as mean ± SD of three independently repeated experiments. Time (h) represents hours of incubation. Doubling time (T_d_) is inferred from OD_600_ measurements during the mid-exponential growth (approximately 3 h of incubation). Pearson’s correlation between cell mass and total DNA content per cell is shown as r(1), and the correlation between the cell mass and the *ori:ter* as r(2), respectively. Excluded from correlation analyses were measurements at 10 h of incubation, since these were used as a denominator in the flow cytometry data (i.e., relative cell mass and relative total DNA content). ***, *p* *≤* 0.001; ****, *p* *≤* 0.0001.

In this comparative growth experiment, all isolates were incubated in Lysogeny broth (LB) batch cultures, from where repeated sample measurements were withdrawn hourly for a total duration of 10 h, which spans both exponential and stationary growth phases. From a combination of population of the mean bacterial growth rate measured by *ori:ter* and single-cell analyses by flow cytometry (i.e., cell mass and total DNA content per cell), we were able to demonstrate that the temporal development in growth and the association between growth parameters were similar in all strains (Figure 2). All strains exerted doubling times (T_d_) of approximately 20 min during the mid-exponential phase. These rapid doubling times correlated with a large cell mass, as well as both high total DNA content per cell and high *ori:ter* ratio. All parameters in accordance with the presence of bacterial cells grew with overlapping replication cycles [28]. After three hours of incubation growth rates gradually declined, since the bacterial populations approached the stationary phase due to starvation of nutrients after prolonged propagation [29]. The absence of bacterial growth during the stationary phase, starting from approximately 6 h of incubation, was demonstrated by a constant low cell mass and total DNA content, along with *ori:ter* at its minimum: ~1. The development in the growth rate measured on a population average (*ori:ter*) correlated with the development in cell mass measured by single-cell analyses in all isolates, to the same extent as the development in single-cell total DNA did (Figure 2). There was no significant difference in overall slopes or intercepts (*ori:ter*) between the isolates (*p* > 0.05). Hence, no strain-specific differences in growth dynamics were observed during the controlled growth *in vitro.*

## 3. Discussion

In this study, we aimed at tracking *in situ* bacterial growth rates from patients with *E. coli* bacteria to better understand the growth dynamics taking place during bacterial propagation in human infection. By the use of differential genome quantification measurements (*ori:ter*) successfully inferred from single urine samples in 28 out of 29 patients, we were able to provide snapshots of bacterial growth rate, reporting directly on bacterial physiology, during the course of infection/colonization in the human urinary tract. Repeated snapshots allowed for novel insight into the temporal bacterial growth dynamics in the urinary tract. In all patients where a dynamic readout was available, we observed day-to-day variation in the bacterial growth rate, regardless of whether the patient was classified as having UTI or asymptomatic bacteria. With only a few exceptions, there was overall active bacterial growth (i.e., *ori:ter >* 1) in the urine, which is consistent with a previous report of rapid bacterial growth measured by whole genome sequencing in eight urine samples from females with uncomplicated UTI [15]. Additionally, urine has, in general, been considered a good growth medium for *E. coli*, as it contains a variety of inorganic salts and organic compounds and is regularly replenished by fresh urine production [1]. In the previously mentioned report, however, only eight out of a total 38 urine samples contained sufficient read coverage for sequencing [15], which is an assay that is both expensive and time-consuming. 

From controlled growth experiments (*in vitro*) of randomly selected patient urine *E. coli* isolates, we confirmed that *ori:ter* measured by qPCR is a robust measure of bacterial growth rate in clinical *E. coli* isolates. In this scenario, we chose a rich medium (LB) to demonstrate the isolates’ potential for maximal growth and compare growth dynamics across isolates. We emphasize that these growth conditions do not necessarily recapitulate *in vivo* conditions, but rather serve as *proof-of-concept*. Maximal *ori:ter* levels observed during growth in this rich medium exceeded those observed *in vivo*, which is consistent with previous comparisons between growth rates of *E. coli* ATCC 25922 *in vitro* and *in vivo* during experimental murine peritonitis/septicemia [19], as well as between various clinical *E. coli* isolates *in vitro* and *in vivo* during experimental murine UTI [15]. In a defined medium with a defined inoculation time, the development in growth dynamics followed a predictable pattern: growth rates were high during early hours of propagation, after which they gradually decreased as the population size increased and entered stationary phase due to nutrient starvation. In human bacteriuria, however, the inoculation time is undefined, and the growth conditions in the urine might be subject to host-specific factors. 

There was no strain-specific variability during controlled growth *in vitro* that could explain the variability in the bacterial growth rate observed between patients. All isolates demonstrated similar growth dynamics under controlled conditions. Consequently, the variability in *ori:ter* ratios observed during human infection rather reflects complex host-pathogen interaction, and/or natural growth variations. Unfortunately, our study population was too heterogenous to perform meaningful analysis to identify a single host parameter to explain this variability. 

*ori:ter* levels in urine samples collected after induction of antibiotic therapy may reflect antibiotic treatment effect or failure. This notably concerns agents targeting nucleic acid synthesis, as observed with trimethoprim in two patients. This observation is in agreement with a previously reported slow-down of replication kinetics during multi-fork replication (and, hence, increase the *ori:ter* ratio) due to experimental nucleotide pool reduction by trimethoprim [30]. The same effect is also observed in ciprofloxacin, which is an antibiotic targeting chromosome replication by inducing replication fork arrest [5]. We observed overall reduction in *ori:ter* (if not total elimination of bacteria) following β-lactam or aminoglycoside therapy, which is suggestive of the treatment effect. This is consistent with previously reported *in vitro* and *in vivo* (experimental murine infection) observations [5]. 

We emphasize that observations from the present study are descriptive. In this case, antibiotic therapy was managed by the clinician and guided by combined clinical and paraclinical findings. Most patients included in the study received combination therapy and/or changing antibiotic regimens. We have, however, demonstrated the successful translation of a robust, inexpensive, and easily available means to measure the *in situ* bacterial growth rate from a single biological sample into the clinical practice, which allows for novel insight into bacterial growth dynamics during a human infection. It is also clear that host factors and treatment regimens must be accounted for in order to study the potential relationship between the growth rate and disease trajectory. 

There is a potential for pursuit in a larger, more homogenous cohort of patients with UTI receiving antibiotic monotherapy to confirm the role of *ori:ter* measurements in evaluation of the treatment effect. Additionally, the fact that the bacterial growth rate in the human urinary tract is a dynamic entity underscores the importance of testing the antibiotic effect as a function of the pretreatment bacterial growth rate in human infections. This could have implications for future antibacterial treatment strategies.

## 4. Materials and Methods 

### 4.1. Study Population and Strain Collection

From 1 January 2018 to 30 May 2018, we identified patients who had significant quantities (≥10^3^ CFU/mL) of *E. coli* cultured from urine samples sent to The Department of Clinical Microbiology at Copenhagen University Hospital, Herlev, Denmark. Eligibility for enrollment were adult inpatients at the hospital’s Department of Infectious Diseases, Dept. of Gastroenterology, and Dept. of Geriatrics or Dept. of General Medicine, who had had the urine sample taken no more than one day prior to *E. coli* identification. Following informed consent, a daily urine sample for up to another three consecutive days was provided from each patient during hospitalization. The study population included patients both with and without symptoms of urinary tract infection (UTI). All urine samples were kept at 4 °C after collection.

### 4.2. Research Ethical Approvals

The Danish Regional Committee on Health Research Ethics (H-17027763) and the Danish Data Protection Agency granted approvals for this study. 

### 4.3. Bacterial Identification and Susceptibility Testing

All urine samples routinely sent to the Department of Clinical Microbiology for culture were handled according to standard laboratory practice. Culture for identification and antimicrobial susceptibility testing was performed on Chrom (Brillance UTI agar; CM949, Oxoid, Basingstoke, UK)—Columbia 5% (Difco Columbia Blood agar Base + 5% horse blood, 279240, BD) bi-plates and disk diffusion test, according to the European Committee on Antimicrobial Susceptibility Testing (EUCAST) standards [31], on Mueller-Hinton agar plates, respectively. Species identification was performed by MALDI-TOF, except for the presentation of typical red colonies on Brilliance UTI agar combined with Cefpodoxime susceptibility. These isolates would be directly identified as *E. coli.* All follow-up urine samples were tested for regrowth of yellow colonies by plating 50 µL in duplicate on bromothymol lactose blue agar plates incubated overnight at 37 °C. 

### 4.4. In Vitro Growth Experiment 

For evaluation of possible strain-specific variation in bacterial growth dynamics, we randomly selected five of the included *E. coli* isolates (# 1, 6, 10, 16, and 29) for comparison of growth in Lysogeny Broth (LB) batch cultures. *E. coli* ATCC 25922 was run in parallel as a control. In this study, an overnight liquid culture of each strain was diluted 1:10,000 into fresh media and grown with shaking 160 rpm at 37 °C. Growth was observed by repeated measurements of optical density at 600 nm (OD_600_). Samples for qPCR analysis and flow cytometry were withdrawn hourly from 2 to 10 h of incubation. All samples were immediately set on ice and fixed by pelleting 1 mL of culture by 5 min centrifugation at 150,000× *g*, after which bacterial cells were re-suspended in 100 µL 10 mM Tris pH 7.4 and 900 µL 77% Ethanol, and then kept at 4 °C until application in downstream analyses. The experiment was independently repeated three times.

### 4.5. Whole-Genome Sequencing and Data Analysis

Bacterial DNA from the same five randomly selected isolates were extracted from liquid cultures and sequenced using the Illumina HiSeq4000 platform, according to the manufacturer’s protocol. *De novo* assembly was done with Unicycler software [32] and contigs greater than 500 base pairs were used to estimate genome size with QUAST: quality assessment tool for genome assemblies [33]. Species identification, *fim*- and serotyping were performed using publicly available web tools from the Center for Genomic Epidemiology (www.genomicepidemiology.org) [34,35,36]. Sequenced genomes are made publicly available by SRA accession number PRJNA523308. 

### 4.6. Flow Cytometry 

Flow cytometry was performed on samples withdrawn from *in vitro* experiments, as previously described [37], using an Apogee A10 instrument. On average, 30,000 cells were analyzed per sample. Measurements of cell mass and total DNA content per cell were recorded and expressed as relative to the cell mass and total DNA content per cell, respectively, of the same strain during the late stationary phase (i.e., the sample collected after 10 h of propagation). 

### 4.7. Real-Time Quantitative PCR (qPCR)

Bacterial DNA from urine samples, concentrated by pelleting of the total amount of urine available (up to 10 mL), was purified for qPCR using QIAamp DNA Mini Kit (51304, Qiagen, Sollentuna, Sweden), according to the manufacturer’s instructions. Fixed samples from the *in vitro* experiments were prepared for qPCR by pelleting by centrifugation, which is followed by re-suspension in serial dilutions of sterile DNA/RNA free water. 

qPCR was performed as previously reported [19], using primers amplifying genes within or in close proximity to *oriC* and *terC*, respectively. Due to a minor variation in amplification efficiencies of the two amplicons in some of the clinical isolates, we tested two primer pair combinations in parallel in each bacterial isolate. The primer pair combination yielding the most optimal amplification efficiency of both amplicons was chosen for analysis in the respective bacterial isolate. *Ori* primer pair #1 and *ori* primer pair #2 were used for partial amplification of the inter-region between *gidA* and *mioC* within the *oriC* region [38], and for partial amplification of the highly conserved *gidA* gene located immediately leftwards of *oriC* [39], respectively (Table S2). *Ter* primer pair #1 and *ter* primer pair #2 were used for partial amplification of *ynfD/ynfE* within the *terC* region [38], and for partial amplification of the *dcp* gene located in close proximity to *terC* [39], respectively (Table S2). Primer specificity for *E. coli* was verified using NCBI BLAST (https://blast.ncbi.nlm.nih.gov) [40]. For *ori* and *ter* primer pair #2, there was 100% identity with *E. coli* only, whereas for *ori* and *ter* primer pair #2, there was 100% identity with both *E. coli* and Shigella spp. This was considered acceptable, since Shigella is no common uropathogen and was not identified in any of the urine samples included.

Expression of *oriC* relative to *terC* (i.e., *ori:ter*) was calculated using comparative cycle threshold (Ct) analysis adjusted according to the exact amplification efficiency for each amplicon (Pfaffl method) [41]. Amplification efficiencies were calculated for both primer pair combinations by linear regression analysis of Ct-values from serial dilutions of every strain grown into a late stationary phase. Amplification efficiencies of 90% to 105% and r^2^ of > 0.98 were considered acceptable. 

A fixed sample of the relevant strain grown into late stationary phase, where the bacterial population would be expected to have an *ori:ter* corresponding to 1, was used for normalization in every cycling run. Each biological replicate was analyzed by three technical replicates in each cycling run, and the mean Ct value of the technical triplicates was used to calculate the *ori:ter.* DNA/RNA-free water was used as a negative control template in each run. Correct qPCR amplification was verified by gel electrophoresis.

### 4.8. Statistical Analyses

Statistical significance in nonparametric data was evaluated by the Mann-Whitney *U* test. Correlation by Pearson’s correlation coefficient. Linear regression analysis was used to compare slopes and intercepts. A two-tailed *p*-value of < 0.05 was considered significant. The GraphPad Prism version 7 (GraphPad Software, USA) was applied for statistical analysis and illustration.

## 5. Conclusions 

In this study, we were able to provide snapshots of bacterial growth rate in *E. coli*, reporting directly on bacterial physiology, during the course of infection/colonization in the human urinary tract by the use of differential genome quantification measurements (*ori:ter*) successfully inferred from single urine samples in 28 out of 29 patients. Growth rates measured were indicative of effect of antibiotic treatments of the patients. The fact that the bacterial growth rate in the human urinary tract is a dynamic entity underscores the importance of testing the antibiotic effect as a function of the pretreatment bacterial growth rate in human infections.

## Figures and Tables

**Table 1:** Characteristics of participants.

Characteristic	Value
Total number of participants	29
Median age, years (range)	79 (29–99)
Sex, number female (%)	22 (76)
Symptoms and/or other signs of urinary tract infection (UTI), number of participants (%) *	20 (69)
Median duration of symptoms, days (range) †	1 (1–14)
Urethral catheter before collection of day 0 urine specimen, number of participants (%)	11 (38)
*E. coli* bacteraemia, number of participants (%)	5 (17)

* Signs and symptoms included dysuria, fever, general malaise, lower abdominal pain, and/or urinalysis test strip yielding positive results for leucocytes and/or nitrite. † Only patients who were able to quantify the duration of symptoms of urinary tract infection were included in this record.

## Supplementary Materials

The following are available online at https://www.mdpi.com/2079-6382/8/3/92/s1. Table S1: Overview of all included urine samples, patient characteristics, and bacterial growth rates. Table S2: Primers used in qPCR.
