# Supplementary material for: Growth Rate of Escherichia coli During Human Urinary Tract Infection: Implications for Antibiotic Effect
Source: Antibiotics (Basel). 2019 Jul 12;8(3):92. doi: 10.3390/antibiotics8030092 (PMC6783841; doi:10.3390/antibiotics8030092)
Supplement: Supplementary file 1 [file antibiotics-08-00092-s001.pdf]

antibiotics

*Supplementary Materials*

# Growth rate of *Escherichia coli* during Human Urinary Tract Infection: Implications for Antibiotic Effect

**Maria Schei Haugan [1,2], Frederik Boëtius Hertz [3], Godefroid Charbon [2], Berivan Sahin [1], Anders Løbner-Olesen [2,*] and Niels Frimodt-Møller [1,*]**

[1] Department of Clinical Microbiology, Rigshospitalet, 2100, Copenhagen, Denmark
[2] Department of Biology, University of Copenhagen, 2100, Copenhagen, Denmark
[3] Department of Clinical Microbiology, Herlev Hospital, 2730, Herlev, Denmark
* Correspondence: niels.frimodt-moeller@regionh.dk

**Table S1.** Overview of all included urine samples, patient characteristics and bacterial growth rate.

| Patient no. | Urine sample (-day no.) | *ori:ter* | *E. coli* quantity ($log_{10}$ CFU/ml) | *E. coli* mono-culture (Y/N) | Urethral catheter at urine sampling (Y/N) | Blood culture *E. coli* pos. (Y/N) | Age (yrs) | Sex (F/M) | No. of symptom days[a] | Urine leucocytes ($10^6$/L) | UTI (Y/N)[b] | Antibiotic treatment (susceptibility, if applicable) before relevant urine sampling[c] | Regrowth on selective agar plate (Y/N) |
|---|---|---|---|---|---|---|---|---|---|---|---|---|---|
| 1 | 1-0 | 1.1 | 5 | Y | Y | Y | 78 | F | 1 | 125 | Y[f] | p.o. TMP(S) (prophylaxis) | Y |
| | 1-1 | 1.5 | | | Y | | | | | | | p.o. PMC(R), i.v. TZP(R), i.v. MTZ, i.v. CXM(S) | N |
| | 1-2 | 1.9 | | | Y | | | | | | | i.v. CXM(S) | N |
| 2 | 2-0 | 1.1 | 5 | N | N | N | 50 | F | 4 | 15 | Y | None | Y |
| | 2-1 | NA | | | N | | | | | | | i.v. AMP(R), i.v. GEN(S), p.o. AMX(R) | N |
| | 2-2 | NA | | | N | | | | | | | p.o. AMX(R) | N |
| 3 | 3-0 | 2.2 | 5 | Y | N | N | 79 | F | 1 | 15 | Y | None | Y |
| 4 | 4-0 | 2.2 | 3[e] | N | N | N | 74 | F | NR | NA | N | None | Y |
| | 4-1 | NA | | | N | | | | | | | None | N |
| | 4-2 | NA | | | N | | | | | | | None | N |
| | 4-3 | NA | | | N | | | | | | | None | N |
| 5 | 5-0 | 2.1 | 5 | Y | N | Y | 91 | F | 1 | 125 | Y[f] | None | Y |
| | 5-1 | NA | | | N | | | | | | | p.o. MEC(S), i.v.AMP(R), GEN(S) | N |
| | 5-2 | NA | | | N | | | | | | | i.v. TZP(S) | N |
| | 5-3 | NA | | | N | | | | | | | i.v. TZP(S) | N |
| 6 | 6-0 | 1.4 | 5 | Y | Y | N | 79 | F | 1 | NA | Y | None | Y |
| | 6-1 | 3.9 | | | Y | | | | | | | p.o. TMP(S), CLR | Y |
| | 6-2 | 3.4 | | | Y | | | | | | | p.o. TMP(S), CLR | Y |
| | 6-3 | NA | | | N | | | | | | | p.o. TMP(S), CLR | N |
| 7 | 7-0 | 3.0 | 5 | Y | N | N | 29 | F | 14 | 70 | Y | None | Y |
| | 7-1 | NA | | | N | | | | | | | i.v. AMP(S), GEN(S) | N |
| | 7-2 | NA | | | N | | | | | | | i.v. AMP(S), GEN(S) | N |
| 8 | 8-0 | 1.4 | 5 | Y | N | NA | 88 | F | NR | 70 | N | None | Y |
| | 8-1 | 3.4 | | | N | | | | | | | None | Y |
| 9 | 9-0 | 1.7 | 5 | Y | Y | Y | 92 | F | NA | 70 | Y[f] | None | Y |
| | 9-1 | 1.2 | | | Y | | | | | | | i.v. AMP(R), GEN(S), i.v. PEN | Y |
| | 9-2 | 1.1 | | | Y | | | | | | | i.v. TZP(S) | Y |
| | 9-3 | 1.2 | | | Y | | | | | | | i.v. TZP(S) | Y |
| 10 | 10-0 | 1.1 | 3 | Y | N | N | 63 | F | NR | NA | N | i.v. PEN | Y |
| | 10-1 | 1.7 | | | N | | | | | | | i.v. PEN | Y |
| | 10-2 | 1.6 | | | N | | | | | | | p.o. AMC(S) | N |
| | 10-3 | 1.6 | | | N | | | | | | | p.o. AMC(S) | N |
| 11 | 11-0 | 2.8 | 5 | N | N | N | 69 | M | 4 | 70 | Y | None | Y |
| | 11-1 | NA | | | N | | | | | | | p.o. MEC(R) | Y |
| | 11-2 | 1.8 | | | N | | | | | | | i.v. TZP(S) | Y |
| 12 | 12-0 | 1.2 | 5 | N | N | NA | 99 | F | 3 | 70 | Y | None | Y |
| | 12-1 | 1.7 | | | N | | | | | | | None | Y |
| 13 | 13-0 | 1.7 | 5 | N | Y | N | 78 | F | 1 | < 15 | Y | None | Y |
| 14 | 14-0 | 1.6 | 3 | N | Y | N | 56 | F | NR | NA | N | i.v. GEN(S) (prophylaxis) | Y |
| 15 | 15-0 | 2.6 | 4[e] | Y | N | NA | 56 | F | 0 | NA | Y | i.v. TZP(S) (prophylaxis) | Y |
| | 15-1 | NA | | | Y | | | | | | | None | N |
| 16 | 16-0 | 1.0 | 5 | Y | Y | N | 84 | F | NA | 500 | Y | None | Y |
| | 16-1 | NA | | | Y | | | | | | | i.v. TZP(S) | Y |
| | 16-2 | NA | | | Y | | | | | | | i.v. TZP(S) | Y |
| | 16-3 | NA | | | Y | | | | | | | p.o. MEC(R), i.v. CXM(S) | N |
| 17 | 17-0 | 1.1 | 5 | N | Y | N | 84 | M | 1 | 70 | Y | None | Y |
| 18 | 18-0 | 1.0 | 5 | Y | N | NA | 82 | F | NA | 70 | Y | None | Y |
| | 18-1 | 2.1 | | | N | | | | | | | p.o. TMP(R) | Y |
| 19 | 19-0 | 2.7 | 5 | Y | N | N | 78 | M | NR | 70 | N | None | Y |
| | 19-1 | 1.1 | | | Y | | | | | | | i.v. TZP(S), MTZ | Y |
| 20 | 20-0 | 1.3 | 5 | Y | Y | N | 69 | F | NR | < 15 | N | None | Y |
| | 20-1 | 2.8 | | | Y | | | | | | | i.v. TZP(S) | N |
| | 20-2 | 1.0 | | | Y | | | | | | | i.v. TZP(S) | N |
| 21 | 21-0 | 1.0 | 5 | N | Y | N | 93 | M | 14 | 500 | Y | None | Y |
| | 21-1 | 1.2 | | | Y | | | | | | | None | Y |
| | 21-2 | 1.5 | | | Y | | | | | | | None | Y |
| 22 | 22-0 | 1.2 | 5 | Y | N | N | 91 | F | 1 | < 15 | Y | None | Y |
| | 22-1 | 1.0 | | | N | | | | | | | p.o. MEC(S) | N |
| 23 | 23-0 | NA | 5 | N | N | Y | 94 | F | NA | 500 | Y[f] | None | Y |
| | 23-1 | NA | | | N | | | | | | | i.v. AMP(R), GEN(S) | Y |
| 24 | 24-0 | 2.2 | 5 | Y | N | NA | 88 | M | 4 | 500 | Y | None | Y |
| | 24-1 | 2.0 | | | N | | | | | | | None | Y |
| 25 | 25-0 | 1.8 | 5 | N | N | N | 60 | F | 2 | 70 | Y | None | Y |
| | 25-1 | 1.9 | | | N | | | | | | | i.v. AMP(R), GEN(S) | Y |
| 26 | 26-0 | 1.6 | 5 | N | N | N | 61 | M | NR | NA | N | None | Y |
| | 26-1 | 2.1 | | | N | | | | | | | None | Y |
| 27 | 27-0 | 2.2 | 5 | Y | Y | Y | 83 | F | 2 | 15 | Y | None | Y |
| | 27-1 | NA | | | Y | | | | | | | i.v. TZP(S), MTZ, CLR | N |
| 28 | 28-0 | 1.4 | 5 | N | Y | N | 82 | M | NA | 70 | NA | None | Y |
| | 28-1 | 1.3 | | | Y | | | | | | | i.v. TZP(R), MTZ, CLR | Y |
| 29 | 29-0 | 1.6 | 5 | N | N | N | 88 | F | NR | NA | N | None | Y |
| | 29-1 | NA | | | N | | | | | | | p.o. CLR, p.o. MXF(S) | N |

[a] No of symptom days (if any) before collection of day 0 urine sample. [b]Patient defined as having UTI or not by the clinician (based on a combination of symptoms, clinical and paraclinical findings). [c]Any antibiotic given during observation period (for either UTI or other infection). [d]Patient recovery within observation period as defined by the clinician (based on a combination of symptoms, clinical and paraclinical findings). [e]ESBL positive isolate. [f]Urosepsis. NA: not available; NR: not relevant (no UTI symptoms). AMX: amoxicillin; AMC: amoxicillin-clavulanic acid; AMP: ampicillin; CXM: cefuroxime; CLR: clarithromycin; GEN: gentamicin; MTZ: metronidazole; MOX: moxifloxacin; PEN: penicillin; TZP: piperacillin-tazobactam; PMC: pivmecillinam; TMP: trimethoprim.

**Table S2.** Primers used in qPCR.

| Name | Primer Sequence (5'-3') | Amplicon Size (bp) | Reference |
|---|---|---|---|
| *ori* FW#1 | CTGTGAATGATCGGTGATCC | 134 | [37] |
| *ori* RV#1 | GTGGATAACTCTGTCAGGAAGCTTG | | |
| *ter* FW#1 | AACTACGCGGGAAATACCC | 175 | [37] |
| *ter* RV#1 | TATCTTCCTGCTCAACGGTC | | |
| *ori* FW#2 | CGCAACAGCATGGCGATAAC | 164 | [38] |
| *ori* RV#2 | TTCGATCACCCCTGCGTACA | | |
| *ter* FW#2 | TCAACGTGCGAGCGATGAAT | 144 | [38] |
| *ter* RV#2* | TTGAGCTGCGCTTCATCGAG | | |

* Original primer modified by 2 bp changes for optimized use in pathogenic *E. coli.*

© 2019 by the authors. Licensee MDPI, Basel, Switzerland. This article is an open access article distributed under the terms and conditions of the Creative Commons Attribution (CC BY) license (http://creativecommons.org/licenses/by/4.0/).
